# Digital Health in Psychiatric Aftercare – Evaluation of the App Flowzone for Bridging Waiting Times in Treatment of Depression

**DOI:** 10.1192/j.eurpsy.2025.1449

**Published:** 2025-08-26

**Authors:** M. Ziegenbein, V. Rößner-Ruff, C. A. Penkov, K. Friedrich, J. Krieger

**Affiliations:** 1 Psychiatry and Psychotherapy, Wahrendorff Klinikum, Sehnde, Germany

## Abstract

**Introduction:**

Since the risk of relapse is particularly high after completion of acute treatment of depression until remission, the guidelines recommend maintenance therapy or continuation over several months and subsequent relapse prevention. In contrast, average waiting time for further outpatient treatment after a part-time inpatient stay is 19.9 weeks in Germany. Digital health technologies can help to support patients at the vulnerable interface when changing health care sectors from part-time inpatient treatment to further outpatient treatment and contribute to a successful change of health care sectors.

**Objectives:**

Flowzone is a digital communication platform in which therapists and patients can stay in contact after completion of part-time inpatient treatment. Individualized therapy content can be shared with patients in weekly plans and communication can be maintained via the chat function. The aim of the current study is to investigate whether Flowzone enables continuity despite long waiting times when health care sectors changes from part-time inpatient to outpatient care.

**Methods:**

In a longitudinal intervention study depressive symptoms (BDI-II) and quality of life (WHOqoL-Bref) will be assed at the end of part-time inpatient treatment (t1) and 8 weeks after end of treatment (t2). Participants diagnosed with depression will be recruited in a day clinic with treatment focus of men of a psychiatric psychotherapeutic specialist hospital in Lower Saxony (Germany). Participants of control group (CG, *n* = 11) will take part in the local 8 week aftercare groups of the psychiatric hospital. Participants of intervention group (IG, *n* = 21) will take part in the digital aftercare with Flowzone. Furthermore subjective benefit, of IG will be measured. In addition, the subjective benefit and user experiences of IG will be measured.

**Results:**

Mean BDI scores of IG are t1 = 14 vs. t2 = 15 and of CG t1 = 9. Mean values of IG for WHOqoL-Bref domains are general quality of life t1= 77/ t2 = 73; physical health t1= 71/ t2 = 68; psychological health t1 = 59/ t2 = 55; social relationships t1 = 65/ t2 = 57; environment t1 = 77/ t2= 71. Further inferential statistical analysis and group comparisons will be reported when date are available.

**Conclusions:**

Results might suggest that depressive symptoms and quality of life could be stabilized with the use of Flowzone 8 weeks after part-time inpatient treatment. Further data collection will allow statistical comparisons to CG. Inclusion of the CG with standard follow-up in an outpatient setting is needed. Flowzone could help to bridge the gap in treatment when patients switch health care sectors and experience a gap between part-time inpatient care and further outpatient care.

**Disclosure of Interest:**

None Declared

